# Nomogram for Deep Vein Thrombosis Prediction Post‐Endovascular Thrombectomy in Acute Ischemic Stroke: A Retrospective Multicenter Observational Study

**DOI:** 10.1111/jocn.17786

**Published:** 2025-04-18

**Authors:** Li Han, Teng‐Wei Pan, Li‐Li Yang, Wei‐Yang Qian, Xiao‐Ping Xu, Feng Wang, Wei‐Zhen Wang, Yang Liu, Wei‐Ying Yang

**Affiliations:** ^1^ Department of Neurology Taizhou Hospital of Zhejiang Province, Affiliated to Wenzhou Medical University Linhai Zhejiang China; ^2^ Department of Neurosurgery Taizhou Hospital of Zhejiang Province, Affiliated to Wenzhou Medical University Linhai Zhejiang China; ^3^ Department of Nursing Taizhou Hospital of Zhejiang Province, Affiliated to Wenzhou Medical University Linhai Zhejiang China; ^4^ Department of Neurology Saarland University Homburg Germany

**Keywords:** acute ischemic stroke, deep vein thrombosis, endovascular thrombectomy, nomogram

## Abstract

**Background:**

Deep vein thrombosis (DVT) is a frequent complication following endovascular thrombectomy (EVT) in patients with acute ischaemic stroke (AIS), potentially leading to fatal pulmonary embolism (PE). Identifying patients early at high risk for DVT is clinically important. This study developed and validated a nomogram combining laboratory findings and clinical characteristics to predict the risk of lower‐extremity DVT after EVT in patients with AIS.

**Methods:**

This retrospective multicentre observational study was conducted in two tertiary hospitals in China, enrolling 640 patients who underwent ultrasonography for DVT diagnosis within 10 days following EVT. Data on medical history, examination and laboratory results were collected for logistic regression analyses to develop a DVT risk nomogram.

**Results:**

Logistic regression analyses identified critical predictors of DVT: lower limb National Institutes of Health Stroke Scale (NIHSS) score ≥ 2, elevated D‐dimer levels (≥ 1.62 mg/L) and prolonged puncture‐to‐recanalization time (PRT ≥ 66 min). The nomogram demonstrated good discriminative ability (AUC 0.741–0.822) and clinical utility across internal and external validation cohorts. Additionally, the presence of DVT was significantly associated with reduced functional independence at 90 days post‐EVT, highlighting the negative impact of DVT on patient recovery (OR = 3.85; 95% CI: 2.18–6.78; *p* < 0.001).

**Conclusion:**

The study provides a practical clinical tool for early detection and intervention in patients with AIS at high risk for DVT following EVT. Early identification and intervention may help improve outcomes in patients with AIS undergoing EVT.

**Relevance to Clinical Practice:**

This nomogram helps in the early detection and proactive management of DVT in AIS patients, which can reduce severe complications and improve patient recovery outcomes.

**Patient or Public Contribution:**

No patient or public contributions were involved in this study due to its retrospective design, where data were utilised from existing medical records without direct patient interaction.

**Summary**

- Deep vein thrombosis is a common complication of acute ischaemic stroke, especially in stroke patients undergoing endovascular thrombectomy, leading to poor functional recovery and even a fatal outcome.
- A National Institutes of Health Stroke Scale score ≥ 2 for the lower extremities, an elevated D‐dimer level ≥ 1.62 mg/L and a prolonged time from puncture to recanalisation ≥ 66 min during thrombectomy are independent predictors of the development of deep vein thrombosis.
- A nomogram integrating all three of the above predictors has provided a practical clinical tool for early identification of stroke patients at high risk of deep vein thrombosis.

## 1 Background

Ischemic stroke is a major cause of death and disability worldwide, contributing significantly to the global health burden (Collaborators 2024). In 2021, there were approximately 7.8 million new cases globally, indicating a continuing rise in stroke incidence (Hou et al. 2024). Stroke survivors, particularly in the acute phase, are at a higher risk of deep vein thrombosis (DVT) due to prolonged immobility and restricted movement, making it a common complication (Han et al. 2023; Kelly et al. 2001). Duplex ultrasonography has shown that the prevalence of DVT in lower extremities in patients with ischemic or haemorrhagic stroke ranges from 11.5% to 22.1% (Li et al. 2023, 2017; Mori et al. 2021; Wang et al. 2019). In our recent study, even 27.3% of patients with ischemic stroke after endovascular thrombectomy (EVT) were found to have DVT in the lower extremities, although they were asymptomatic (Han et al. 2023).

DVT not only increases the risk of long‐term disability but also carries the potential for fatal pulmonary embolism (Kelly et al. 2001; Pongmoragot et al. 2013). Isolated distal DVT is thought to be benign compared to proximal DVT and pulmonary embolism; however, calf DVT has been reported to propagate to the thigh in up to 20% of cases (Philbrick and Becker 1988). EVT has significantly improved outcomes in AIS due to large vessel occlusion (Goyal et al. 2016). Although the risks of DVT in stroke patients are well documented, the American Heart Association/American Stroke Association (AHA/ASA) guidelines provide limited guidance on managing DVT following EVT (Powers et al. 2019). EVT carries unique risks, including the potential for an increased incidence of DVT due to factors, such as prolonged immobilisation and endothelial damage (Han et al. 2023). The non‐specific nature of venous thromboembolism symptoms complicates diagnosis, particularly in patients with post‐stroke speech, cognitive or psychiatric impairments, which are further exacerbated by the timing constraints of ultrasound imaging. These challenges may lead to the underdiagnosis or delayed diagnosis of DVT, particularly after EVT (Kelly et al. 2001).

Our study aimed to develop and validate a nomogram that integrates clinical characteristics and laboratory findings to predict the risk of lower‐extremity DVT post‐EVT in patients with AIS, which could refine post‐EVT care protocols and promote improved outcomes by facilitating early identification and management of patients with DVT.

## 2 Methods

### 2.1 Patients and Study Design

This retrospective observational cohort study was performed at Taizhou Hospital of Zhejiang Province, affiliated to Wenzhou Medical University and Enze Hospital, Taizhou Enze Medical Center. It analysed patients with AIS who received EVT from January 2020 to December 2022. Patients treated at Taizhou Hospital constituted the training and internal validation cohorts, whereas those at Enze Hospital formed the external validation cohort.

Patients were included in the study based on the following criteria: (a) ≥ 18 years of age; (b) diagnosis of acute ischemic stroke, confirmed via computed tomography (CT) or magnetic resonance imaging (MRI), aligning with the 2019 American Heart Association Stroke Guidelines (Powers et al. 2019); and (c) diagnosis of large vessel occlusion through digital subtraction angiography or CT angiography. Exclusion criteria encompassed: (a) absence of ultrasound; (b) mortality before ultrasound; (c) history of VTE; (d) unsuccessful EVT recanalisation, assessed by modified thrombolysis in cerebral infarction (mTICI) with a threshold of 2b or higher for success (Nogueira et al. 2018); (e) congenital absence of lower limbs; and (f) loss to follow‐up.

### 2.2 Data Collection

Three of the authors meticulously collected comprehensive patient data, including previous medical histories, examination results, laboratory findings and diagnoses from the electronic medical records at Taizhou Hospital and Enze Hospital. Guided by previous research (Han et al. 2023; Liu et al. 2014; Wang et al. 2019) and clinical expertise, we identified potential factors for our predictive model. Extracted clinical information included age, gender, atrial fibrillation, hypertension, diabetes, smoking status, history of malignant tumours, infections (pulmonary and urinary tract), use of mechanical ventilation, central venous catheter placement, anaesthesia type (conscious sedation or general anaesthesia), pre‐treatment with intravenous thrombolysis, intracranial occlusion site (anterior or posterior circulation), puncture‐to‐recanalization time (PRT), infarct core volume, time‐to‐maximum (Tmax > 6 s), mismatch volume (for patients who underwent CT perfusion imaging), Trial of ORG 10172 in Acute Stroke Treatment (TOAST) classification (large‐artery atherosclerosis, small‐artery occlusion, cardioembolism, other determined cause and undetermined cause), intra‐ or post‐procedural medications (mannitol, sedative drugs, tirofiban and antithrombotic drugs), laboratory results within 48 h post‐EVT, duration of stay in the intensive care unit (ICU), total and lower limb NIHSS scores 24 h post‐EVT. Univariate logistic regression was applied to all continuous variables. Those with a *p* value less than 0.1 were converted into binary variables, including D‐dimer levels, age and PRT. Using receiver operating characteristic (ROC) curves, optimal thresholds for each variable were identified based on the maximum Youden index. Accordingly, cut‐off values were set at 1.62 mg/L for D‐dimer, 70 years for age and 66 min for PRT, as we did in a recent study (Han et al. 2023). Lower extremity NIHSS scores were divided into categories of ≥ 2 and < 2 for clarity in analysis.

The modified Rankin scale (mRS) score 90 days after EVT was used to assess functional outcomes. Patients who underwent treatment at the Taizhou Hospital of Zhejiang Province were included in the analysis. The patients underwent outpatient or telephonic follow‐up approximately 90 days after EVT (with a margin of ±14 days), during which they were assigned mRS scores. An mRS score of ≤ 2 was considered functionally independent (Nogueira et al. 2018).

### 2.3 Endpoint Ascertainment and Post‐EVT Prevention of DVT

DVT was diagnosed using a complete compression duplex ultrasonography examination using our established protocol (Han et al. 2023) that identified deep venous structures in the patient's legs. Ultrasonography was performed within 10 days post‐EVT for all evaluated individuals.

As indicated in the AHA/ASA guidelines, the benefit of prophylactic heparin dosing in patients with AIS has not been adequately demonstrated (Powers et al. 2019). Patients without hemorrhagic complications received antiplatelet therapy 24 h after EVT. In addition, 16 patients received anticoagulant therapy. Intermittent pneumatic compression (IPC), applied for 18 h daily immediately after EVT, was used without contraindications and continued throughout the ICU stay. IPC was extended to immobile patients on transfer to the general ward.

### 2.4 Statistical Analysis

Statistical analyses for this study were performed using R version 4.2.3 (R Project for Statistical Computing, Vienna, Austria). We used descriptive statistics to summarise the baseline characteristics of the three cohorts; categorical variables were presented as frequencies and proportions, whereas continuous variables were described using means and standard deviations (SD) or medians and interquartile ranges (IQR), depending on the distribution of the data. Univariate logistic regression was used within the training cohort to assess associations between variables and DVT outcomes. Variables showing a significant association (*p* < 0.05) in the univariate analysis were then entered into a multifactorial logistic regression to identify independent risk factors for DVT, using backward elimination based on Akaike's information criterion for model refinement. Regression coefficients were used as weights in the prediction model. In order to evaluate the influence of stroke severity on DVT risk, NIHSS scores were categorised into three groups (mild: 0–4, moderate: 5–15 and severe: > 15). Logistic regression was adjusted for these categories in the training set to ensure the model's robustness across different severity levels. Following multivariate logistic regression analysis, we developed a nomogram to delineate the risk of DVT after EVT. Evaluation of the model included discrimination, calibration and net clinical benefit, with discrimination assessed by the area under the ROC curve and calibration by calibration curves alongside the Hosmer–Lemeshow test. The clinical utility of the nomogram was evaluated using decision curve analysis (DCA), which calculated the net benefit at various threshold probabilities. The net benefit is the proportion of true positives minus the proportion of false positives, and the relative hazard of false‐positive results was standardised, as previously described (Liu et al. 2021). We applied the model to an independent dataset from Enze Hospital, Taizhou Enze Medical Center, to assess the external validity of the model. *p* < 0.05 indicated statistical significance.

### 2.5 Informed Consent and Ethical Approval

The requirement for informed consent was waived due to the retrospective design of the study, which followed paragraph 32 of “World Medical Association Declaration of Helsinki” that informed consent may be waived in research where it is impractical to obtain and where the research involves minimal risk to participants (World Medical Association 2013). The study procedure was also approved by the Institutional Ethics Committees of Taizhou Hospital of Zhejiang Province (registration number: K20181204) and Enze Hospital, Taizhou Anze Medical Center (registration number: K20230415).

## 3 Results

### 3.1 Baseline Characteristics

There were 640 patients enrolled in our study: 417 from Taizhou Hospital (TH) and 223 from Enze Hospital (EH). The participants were between 18 and 95 years old, with a median age of 69 (IQR 60–76). Specifically, the training cohort, the internal validation cohort and the external validation cohort had a median age of 69 (IQR, 60–75), 67 (IQR, 60–73) and 71 (IQR, 61–79) years, respectively, without difference between each two groups (Table 1; *p* > 0.05). The gender distribution also did not differ between these three groups (Table 1; 38.4%, 35.2% and 38.6% female patients in the training, internal and external validation cohorts, respectively; *p* > 0.05).

**TABLE 1:** Baseline characteristics of the training set and validation cohort.

Variables	Training cohort (*n* = 292)	Internal validation cohort (*n* = 125)	External validation cohort (*n* = 223)
DVT, *n* (%)	74 (25.3)	28 (22.4)	49 (23.0)
**Demographic characteristics**			
Age, median (Q1, Q3)	69 (60, 75)	67 (60, 73)	71 (61, 79)
Female gender, *n* (%)	112 (38.4)	44 (35.2)	86 (38.6)
*TOAST subtypes*			
Large‐artery atherosclerosis	145 (49.7%)	72 (57.6%)	127 (57.0%)
Small‐artery occlusion	0	0	0
Cardioembolism	132 (45.2%)	46 (36.8%)	88 (39.5%)
Other determined cause	5 (1.7%)	2 (1.6%)	3 (1.3%)
Undetermined cause	10 (3.4%)	5 (4.0%)	5 (2.2%)
**Risk factors for stroke**			
Atrial fibrillation, *n* (%)	137 (46.9)	44 (35.2)	61 (27.4)
Hypertension, *n* (%)	171 (58.6)	67 (53.6)	135 (60.5)
Diabetes, *n* (%)	63 (21.6)	21 (16.8)	38 (17.0)
Smoking, *n* (%)	91 (31.2)	30 (24.0)	54 (24.2)
**Risk factors for DVT**			
Malignant tumours, *n* (%)	21 (7.2)	6 (4.8)	7 (3.1)
Infections, *n* (%)	171 (58.6)	66 (52.8)	81 (36.3)
Mechanical ventilation, *n* (%)	79 (27.1)	30 (24.0)	33 (14.8)
Central venous catheter, *n* (%)	94 (32.2)	40 (32.0)	36 (16.1)
**Characteristics of EVT**			
*Anaesthesia type*, *n* (%)			
Conscious sedation	224 (76.7)	103 (82.4)	215 (96.4)
General anaesthesia	68 (23.3)	22 (17.6)	8 (3.6)
Pre‐treatment intravenous thrombolysis, *n* (%)	66 (22.6)	39 (31.2)	68 (30.5)
*Intracranial occlusion site*, *n* (%)			
Anterior circulation	236 (80.8)	103 (82.4)	195 (87.4)
Posterior circulation	56 (19.2)	22 (17.6)	28 (12.6)
PRT, median (Q1, Q3)	69 (47, 105)	60 (45, 90)	64 (45, 94)
Valid observations/total sample size	167/292	76/125	146/223
Infarct core volume	2.0 (0.0, 11.5)	2.6 (0.0, 12.0)	4.0 (0.0, 12.8)
Tmax > 6 s	87.0 (51.5, 130.0)	88.5 (45.8, 127.2)	72.5 (39.0, 119.4)
Mismatch volume	77.0 (46.0, 117.0)	75.0 (40.3, 113.0)	64.5 (32.7, 101.0)
**Intra‐ or post‐procedural medications**			
Mannitol, *n* (%)	170 (58.2)	78 (62.4)	123 (55.2)
The sedative drug, *n* (%)	111 (38.0)	41 (32.8)	72 (32.3)
Tirofiban, *n* (%)	143 (49.0)	67 (53.6)	112 (50.2)
*Antithrombotic drugs*, *n* (%)			
Antiplatelet drugs	267 (91.4)	115 (92.0)	202 (90.6)
Anticoagulant drugs	4 (1.4)	4 (3.2)	8 (3.6)
No antithrombotic drugs	21 (7.2)	6 (4.8)	13 (5.9)
**Laboratory findings within 48 h after EVT**			
D‐dimer, median (Q1, Q3)	1.3 (0.6, 2.5)	1.1 (0.7, 2.2)	1.21 (0.6, 2.2)
CRP, median (Q1, Q3)	5.4 (2.2, 16.3)	6.2 (2.2, 18.4)	5.4 (2.5, 13.3)
WBC, median (Q1, Q3)	9 (7.4, 11.3)	9.3 (7.4, 10.7)	8.7 (6.9, 10.3)
NEUT, median (Q1, Q3)	7.3 (5.7, 9.8)	7.4 (6.0, 8.8)	7.0 (5.4, 8.5)
LYM, median (Q1, Q3)	1.0 (0.8, 1.4)	1.0 (0.7, 1.3)	1.0 (0.8, 1.5)
MONO, median (Q1, Q3)	0.4 (0.3, 0.6)	0.4 (0.3, 0.6)	0.4 (0.3, 0.5)
HB, median (Q1, Q3)	127 (113, 137)	128 (119, 138)	126 (115, 137)
PLT, median (Q1, Q3)	191 (156, 219)	190 (158, 229)	193 (154, 233)
Albumin, median (Q1, Q3)	36.1 (33.6, 38.5)	36.0 (33.6, 38.2)	35.7 (33.7, 38.3)
Cr, median (Q1, Q3)	65 (53, 77)	63 (53, 73)	65 (57, 81)
BUN, median (Q1, Q3)	4.9 (3.8, 6.1)	5.1 (3.8, 6.3)	4.4 (3.8, 5.9)
Glucose, median (Q1, Q3)	6.6 (5.6, 8.1)	6.4 (5.3, 8.1)	6.7 (5.6, 7.8)
TG, median (Q1, Q3)	1.01 (0.72, 1.53)	0.97 (0.76, 1.52)	1.12 (0.80, 1.52)
TC, median (Q1, Q3)	4.0 (3.4, 4.7)	4.0 (3.3, 4.8)	4.1 (3.5, 4.8)
HDL, median (Q1, Q3)	1.2 (1.0, 1.4)	1.2 (1.1, 1.4)	1.3 (1.1, 3.2)
LDL, median (Q1, Q3)	2.2 (1.7, 2.7)	2.2 (1.8, 2.7)	2.3 (1.8, 2.9)
Fibrinogen, median (Q1, Q3)	3.0 (2.6, 3.6)	3.0 (2.5, 3.7)	3.0 (2.5, 3.5)
**Clinical findings**			
Extended ICU stays (≥ 3 days), *n* (%)	96 (32.9)	35 (28.0)	37 (16.6)
Lower limb NIHSS score ≥ 2, *n* (%)	163 (55.8)	70 (56.0)	111 (49.8)
NIHSS score 24 h post‐EVT	8 (3, 16)	9 (3, 16)	8 (3, 16)

*Note:* Valid observations/total sample size indicate the number of valid data points for the variables infarct core volume, Tmax > 6 s, and mismatch volume.

Abbreviations: BUN, blood urea nitrogen; Cr, serum creatinine; CRP, C‐reactive protein; DVT, deep vein thrombosis; EVT, endovascular thrombectomy; HB, haemoglobin; HDL, high‐density lipoprotein; ICU, intensive care unit; LDL, low‐density lipoprotein; LYM, lymphocyte; MONO, monocyte; NEUT, neutrophil; NIHSS, National Institutes of Health Stroke Scale; PLT, platelet; PRT, puncture‐to‐recanalization time; TC, total cholesterol; TG, triglyceride; Tmax, time‐to‐maximum; TOAST, Trial of ORG 10172 in Acute Stroke Treatment; WBC, white blood cell.

**FIGURE 1:**
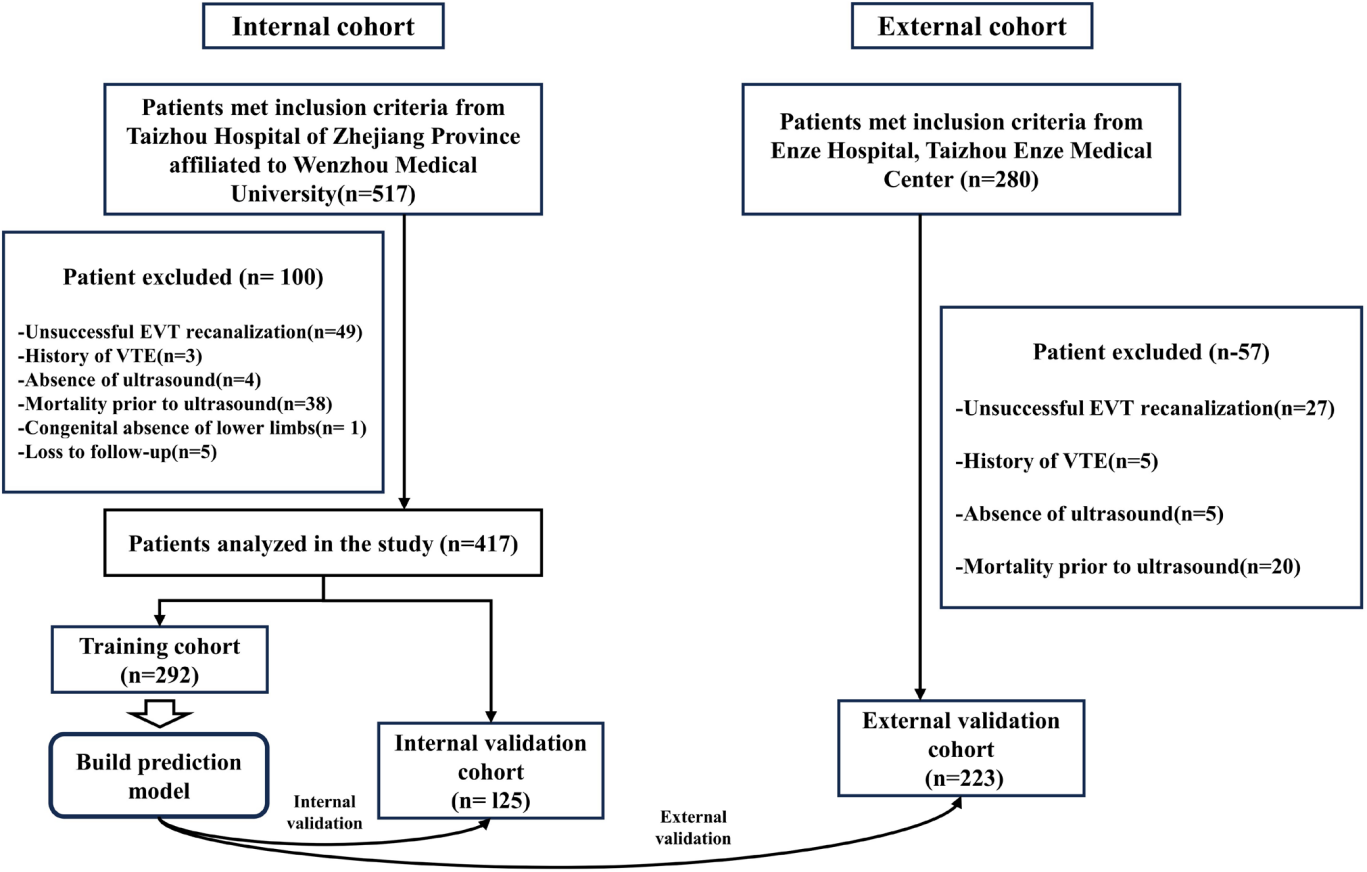
Flow chart of participant selection: (a) Taizhou Hospital of Zhejiang Province affiliated to Wenzhou Medical University cohort; (b) Enze Hospital, Taizhou Enze Medical Center cohort. [Colour figure can be viewed at wileyonlinelibrary.com]

DVT was identified in 151 (23.6%) patients (100 from TH and 51 from EH) with AIS, 143 of whom were diagnosed with distal DVT, with muscular calf veins being the most frequently affected (122 patients [TH: 83 and EH: 39]). In addition, in 87 patients (TH, 59; EH, 28) DVT occurred in one vessel and 49 (TH: 35; EH: 14) patients were diagnosed with bilateral DVT. The total patient population (*n* = 640) was categorised into three cohorts: 417 patients from TH were assigned to the training cohort (*n* = 292) and the internal validation cohort (*n* = 125) by 7:3 randomisation (to develop a predictive model and perform internal validation). All 223 patients from EH were enrolled in the external cohort (to facilitate external validation of the predictive model). A study design flow chart is depicted in Figure 1, whereas baseline characteristics and DVT incidences for each cohort are detailed in Table 1.

### 3.2 Development of a Nomogram Based on the Training Cohort

By using univariate logistic regression in the training cohort, we identified multiple factors significantly associated with an increased DVT risk post‐EVT, which included older age (≥ 70 years), infections (pulmonary and urinary tract), use of mechanical ventilation, central venous catheter placement, anaesthesia type, prolonged PRT (≥ 66 min), use of postoperative mannitol and sedative drugs, absence of postoperative antithrombotic drugs, extended ICU stays (≥ 3 days), elevated D‐dimer levels (≥ 1.62 mg/L), and a lower limb NIHSS score ≥ 2 after EVT (Table 2). All analysed parameters demonstrated *P* values of < 0.05, underscoring their statistical significance.

**TABLE 2:** Univariate and multivariate logistic regression analysis of risk factors for DVT after EVT in the training cohort.

Characteristics	Univariate analysis			Multivariate analysis		
	OR	95% CI	*p*	OR	95% CI	*p*
Female gender	1.526	0.894–2.604	0.121			
Older age (≥ 70 years)	2.212	1.284–3.810	0.004			
TOAST subtypes			0.192			
Large‐artery atherosclerosis	Ref					
Cardioembolism	1.60	0.93–2.75				
Other determined cause	2.45	0.39–15.32				
Undetermined cause	0.41	0.05–3.35				
Atrial fibrillation	1.579	0.929–2.684	0.092			
Hypertension	0.841	0.494–1.432	0.524			
Diabetes	0.900	0.470–1.727	0.752			
Smoking	0.767	0.427–1.378	0.375			
Malignant tumours	0.915	0.323–2.590	0.867			
Infections	3.059	1.673–5.592	0.000			
Mechanical ventilation	2.772	1.58–4.862	0.000			
Central venous catheter	4.542	2.599–7.937	0.000			
Anaesthesia type	1.898	1.054–3.419	0.033			
Pre‐treatment with intravenous thrombolysis	1.138	0.612–2.117	0.682			
Intracranial occlusion site	0.584	0.278–1.227	0.156			
Prolonged PRT (≥ 66 min)	1.873	1.089–3.224	0.023	2.353	1.191–4.649	0.014
Infarct core volume	1.017	0.995–1.038	0.118			
Tmax > 6 s	1.002	0.996–1.007	0.554			
Mismatch volume	1.001	0.994–1.007	0.821			
Mannitol	3.116	1.704–5.696	0.000			
Sedative drug	1.946	1.14–3.323	0.015			
Tirofiban	0.790	0.465–1.342	0.384			
*Antithrombotic drugs*						
Antiplatelet drugs	Ref					
Anticoagulant drugs	1.102	0.113–10.786	0.933			
No antithrombotic drugs	3.637	1.475–8.966	0.005			
Elevated D‐dimer (≥ 1.62 mg/L)	4.752	2.71–8.331	0.000	3.237	1.645–6.367	0.001
WBC	1.019	0.942–1.101	0.643			
CRP	0.998	0.99–1.006	0.688			
NEUT	1.030	0.953–1.113	0.455			
LYM	0.730	0.429–1.241	0.245			
MONO	0.866	0.393–1.906	0.721			
HB	0.999	0.985–1.013	0.867			
PLT	1.001	0.996–1.005	0.795			
Albumin	0.946	0.887–1.009	0.094			
Cr	0.996	0.985–1.008	0.509			
BUN	1.010	0.890–1.146	0.879			
Glucose	0.968	0.860–1.09	0.592			
TG	0.924	0.772–1.105	0.385			
TC	0.910	0.724–1.143	0.416			
HDL	0.822	0.475–1.422	0.483			
LDL	0.843	0.596–1.192	0.333			
Fibrinogen	1.240	0.960–1.603	0.100			
Extended ICU stays (≥ 3 days)	4.682	2.677–8.188	0.000			
Lower limb NIHSS score ≥ 2	12.164	5.34–27.705	0.000	6.247	2.369–16.47	0.000
NIHSS score 24 h post‐EVT	0.99	0.935–1.047	0.714			

*Note:* Data for infarct core volume, Tmax > 6 s, and mismatch volume were available for a subset of patients who underwent CT perfusion imaging.

Abbreviations: BUN, blood urea nitrogen; Cr, serum creatinine; CRP, C‐reactive protein; DVT, deep vein thrombosis; EVT, endovascular thrombectomy; HB, haemoglobin; HDL, high‐density lipoprotein; ICU, intensive care unit; LDL, low‐density lipoprotein; LYM, lymphocyte; MONO, monocyte; NEUT, neutrophil; NIHSS, National Institutes of Health Stroke Scale; PLT, platelet; PRT, puncture‐to‐recanalization time; TC, total cholesterol; TG, triglyceride; Tmax, time‐to‐maximum; TOAST, Trial of ORG 10172 in Acute Stroke Treatment; WBC, white blood cell.

**FIGURE 2:**
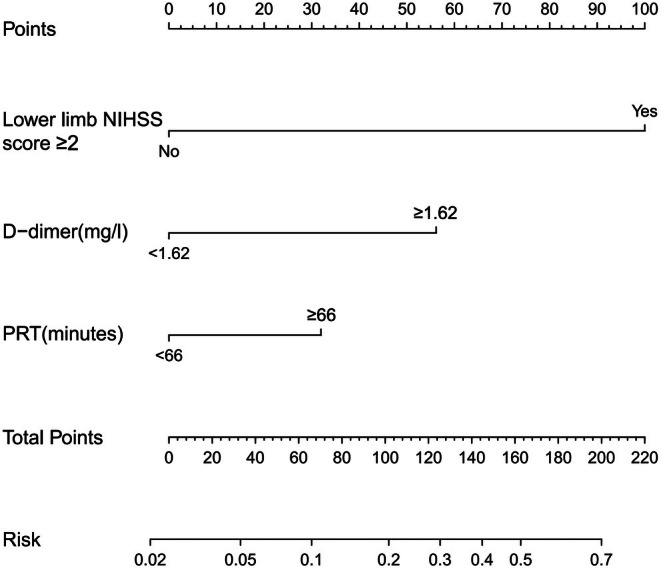
Nomogram for the risk of developing deep vein thrombosis following endovascular thrombectomy. The values of each variable were scored on a scale of 0–100, and the scores for each variable were then summed. This sum is located on the total score axis, from which we can predict the probability of developing deep vein thrombosis risk. Lower limb National Institutes of Health Stroke Scale score ≥ 2 = 100 points, D‐dimer ≥ 1.62 mg/L = 56 points, puncture‐to‐recanalization time ≥ 66 min = 32 points.

By further using multivariate logistic regression analysis, we observed that elevated D‐dimer levels (≥ 1.62 mg/L) (OR = 3.237; 95% CI: 1.645–6.367; *p* = 0.001), lower limb NIHSS score ≥ 2 (OR = 6.247; 95% CI: 2.369–16.47; *p* < 0.001), and prolonged PRT (≥ 66 min) (OR = 2.353; 95% CI: 1.191–4.649; *p* = 0.014) were each independently associated with an increased DVT risk in patients with AIS after EVT (Table 2). These variables were key predictors in our predictive model for assessing DVT risk post‐EVT. The logistic regression formula, −3.6959 + 0.7258 × (prolonged PRT) + 1.2758 × (elevated D‐dimer) + 2.2719 × (lower limb NIHSS score ≥ 2), underpinned the construction of a nomogram (Figure 2) for DVT risk prediction. The nomogram assigned specific points based on the values of three critical predictors to determine the risk of DVT: lower limb NIHSS score ≥ 2 is assigned 100 points, elevated D‐dimer (≥ 1.62 mg/L) is assigned 56 points, and prolonged PRT (≥ 66 min) is assigned 32 points. The total points are obtained by summing the points for each variable, which correspond to the predicted risk of DVT on the nomogram.

### 3.3 Performance of the Nomogram in the Training Cohort

**FIGURE 3:**
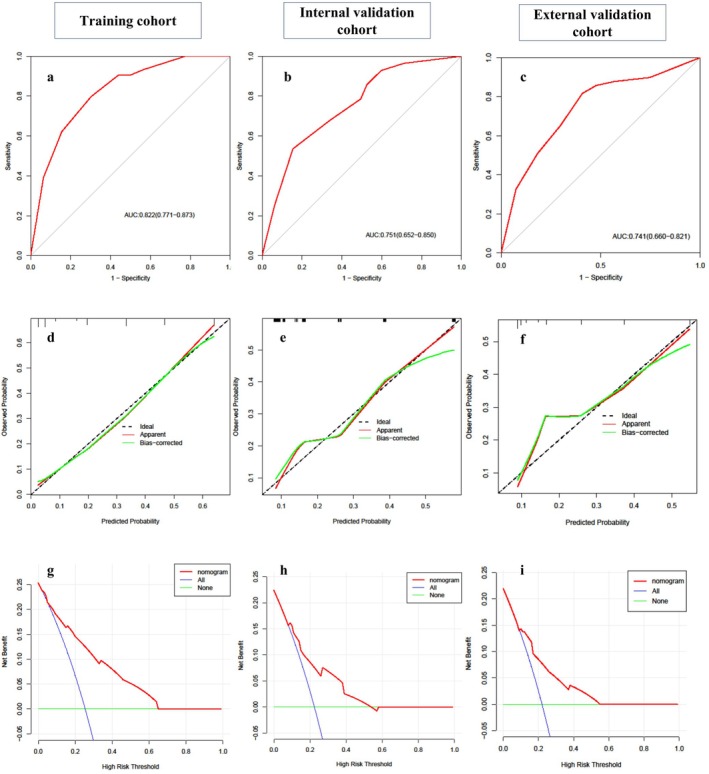
Comprehensive evaluation of the nomogram across various cohorts. Panels a–c illustrate the receiver operating characteristic (ROC) curves for the training, internal validation and external validation cohorts, respectively, demonstrating the nomogram's discriminative power. Panels d–f display calibration plots for these cohorts, where the nomogram's proximity to the ideal model line indicates accurate prediction. Panels g–i show decision curve analysis for each cohort, assessing the clinical utility of the nomogram. These decision curves highlight the range of threshold probabilities where the nomogram provides a net benefit over standard ‘treat‐all’ or ‘treat‐none’ strategies. [Colour figure can be viewed at wileyonlinelibrary.com]

The DVT‐nomogram demonstrated good discriminatory ability (AUC = 0.822; 95% CI: 0.771–0.873) within the training cohort, as depicted in Figure 3a. Calibration curves, presented in Figure 3d, revealed a good agreement between the nomogram's predictions and the observed outcomes, further validated by a non‐significant Hosmer–Lemeshow test *p* value (*p* = 0.781), suggesting precise calibration. Additionally, DCA, shown in Figure 3g, evaluated the nomogram's clinical utility. DCA for the training cohort showed that applying the nomogram to guide treatment decisions conferred more net benefit than universal treatment or non‐treatment strategies across a threshold probability range of 9% to 65%.

### 3.4 Performance of the Nomogram in the Validation Cohort

In both the internal and external validation cohorts, the nomogram demonstrated excellent discriminatory power, with AUCs of 0.751 (95% CI: 0.652–0.850) and 0.741 (95% CI: 0.660–0.821), respectively (Figure 3b,c). In addition, the nomogram exhibited good calibration in both the internal and external validation cohorts (Figure 3e,f). The Hosmer–Lemeshow test *p* values for the internal and external validation sets were 0.084 and 0.06, respectively. According to the DCA, the net benefit threshold probabilities associated with applying the nomogram ranged from 2% to 65% for the internal validation cohort and 9% to 55% for the external validation cohort (Figure 3h,i).

### 3.5 Performance of the Nomogram in Cohorts Categorised by Total NIHSS Scores

After creating and validating a nomogram to predict DVT in the lower extremities, we investigated how the severity of stroke, as determined by NIHSS total scores 24 h after EVT, affected the predictive power of the nomogram. We further divided the training cohort into three clinically relevant groups (mild: 0–4, moderate: 5–15, severe: > 15) based on the NIHSS total scores. Indeed, the logistic regression analyses showed that the probability of developing DVT increased significantly with higher NIHSS scores (Table S1). In patients with moderate NIHSS scores (5–15) and severe NIHSS scores (> 15), the ORs were 7.88 (95% CI: 3.02–20.55; *p* < 0.001) and 18.56 (95% CI: 6.95–49.54; *p* < 0.001), respectively, compared to patients with mild NIHSS scores (0–4).

Notably, when fitting the logistic regression model to assess the effects of the predictors after accounting for NIHSS categories, we found that the predictors remained stable across different stroke severities (Table S2), with PRT (OR: 2.14; 95% CI: 1.15–4.07; *p* = 0.018), D‐dimer (OR: 3.29; 95% CI: 1.77–6.20; *p* < 0.001), and lower limb NIHSS score (OR: 5.13; 95% CI: 1.60–20.33; *p* = 0.011) as independent risk factors for DVT.

### 3.6 Comparison of the Nomogram With Individual Independent Variables

**FIGURE 4:**
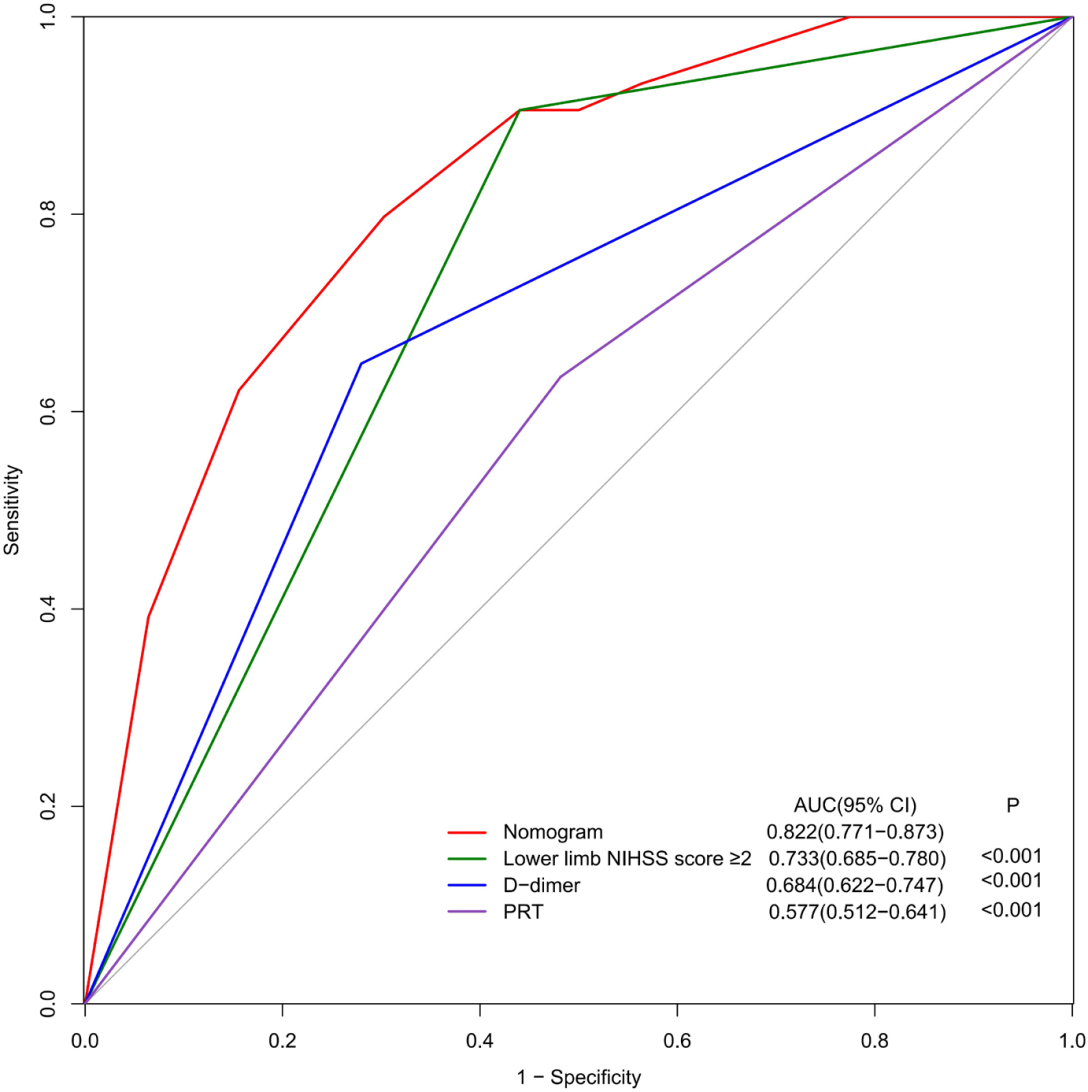
Receiver operator characteristic curves comparing the discriminatory accuracy of the nomogram versus the other variables incorporated in the nomogram alone are shown. *p* values show the areas under the curve (AUCs) for the nomogram versus the AUCs for individual independent variables in the training cohort. [Colour figure can be viewed at wileyonlinelibrary.com]

Figure 4 illustrates the comparative analysis of the ROC curves between the DVT nomogram and the individual independent variables. This analysis shows that the DVT nomogram has superior discriminatory accuracy in predicting DVT in patients with AIS after EVT compared to each independent variable.

### 3.7 Relationship Between Clinical Outcomes and DVT

**FIGURE 5:**
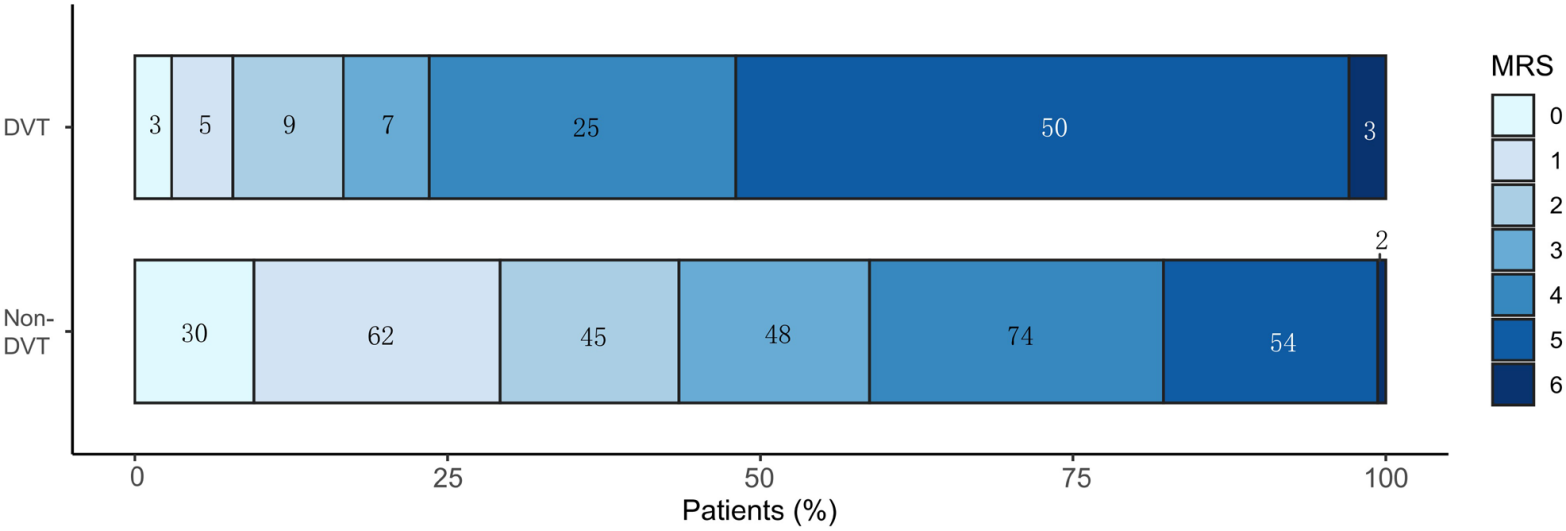
Distribution of modified Rankin scale scores at Day 90 between patients with and without deep vein thrombosis. Scores on the modified Rankin scale range from 0 to 6, with 0 indicating no disability, 3 indicating moderate disability and 6 indicating death. DVT, deep vein thrombosis. [Colour figure can be viewed at wileyonlinelibrary.com]

Finally, we examined 417 patients from Taizhou Hospital for the effects of DVT on disease prognosis. NIHSS scores 24 h after EVT were significantly higher in the DVT group than in the non‐DVT group (8.65 ± 8.52 vs. 14.84 ± 7.51; *p* < 0.001). The mRS results at 90 days showed that only 16.7% (17 of 102) of patients in the DVT group achieved functional independence (mRS ≤ 2), significantly less than 43.5% (137 of 315) in the non‐DVT group (OR = 3.85; 95% CI: 2.18–6.78; *p* < 0.001), as shown in Figure 5. This discrepancy emphasises the detrimental effects of DVT on functional recovery after DVT.

## 4 Discussion

In this study, we developed and validated a nomogram to predict the risk of DVT in patients with AIS after EVT. The nomogram, consisting of a lower extremity NIHSS score ≥ 2, elevated D‐dimers (≥ 1.62 mg/L) and prolonged PRT (≥ 66 min), showed good discriminatory power in predicting DVT. The nomogram can be easily applied in daily clinical practice to identify patients with AIS who are at high risk of DVT after EVT and facilitates early intervention and prevention.

In our analysis, a lower limb NIHSS score ≥ 2 emerged as the strongest predictor of DVT risk after EVT, which is consistent with many other trials and meta‐analyses (Fu et al. 2024; Liu et al. 2014; Wang et al. 2019). By further categorising patients by NIHSS total score (including patients with a score < 5), we demonstrated that lower limb weakness instead of total NIHSS score predominated in predicting DVT, which suggests that our nomogram can also be used to predict DVT in patients with minor stroke. Our study supports the hypothesis that Virchow's triad (hemodynamic changes, endothelial injury, and hypercoagulability) has been established in the veins of lower limbs in AIS patients after EVT, which is the pathological basis for DVT. The loss of muscle contusion impairs the venous pump and leads to a decrease in blood flow or even venous stasis. Prolonged immobility, pressure on the blood vessels (e.g., by lying in one position for a long time) and distension of the vessel walls by the pooling blood may result in localised hypoxia and endothelial cell damage, stimulating endothelial cells to release Willebrand factor and P‐selectin, and recruit platelets and leukocytes (Closse et al. 1997). Neutrophils are the first cells in the blood to react to a stroke. They form neutrophil extracellular traps (NETs) that interact with endothelial cells and platelets, promoting thrombosis in both arteries and veins (Zdanyte et al. 2023). Notably, the negatively charged extracellular DNA surfaces of NETs are a natural procoagulant cofactor in blood coagulation (Kannemeier et al. 2007). Indeed, depletion of neutrophils or disintegration of NETs blocks the propagation of DVT in the mouse model of DVT with restricted blood flow in the inferior vena cava (von Bruhl et al. 2012).

The D‐dimer concentration is the second strongest predictor in our study. In clinical practice, the absence of D‐dimer has often been used to rule out DVT (Kearon et al. 2022). We identified 1.62 mg/L as the optimal cut‐off point for the prediction of DVT after EVT (Han et al. 2023). Interestingly, our cut‐off value is very similar to the cut‐off points (1.52, 1.59 and 1.66 mg/L) in three other studies on the prediction of DVT (Balogun et al. 2016; Harvey et al. 1996; Mori et al. 2021). Since the patients in the three studies did not receive EVT, the high plasma level of D‐dimers predicts DVT in acute stroke patients regardless of EVT.

In the era of thrombectomy, we are the first to add the prolonged duration of EVT (from puncture to recanalization ≥ 66 min) as a third predictor for the development of DVT in AIS patients. Prolonged EVT has been associated with malignant cerebral edema and poor prognosis at 90 days (Li et al. 2022; Ma et al. 2022; Pu et al. 2023). It is still unclear how prolonged EVT promotes DVT. As described above, neutrophil activation is closely associated with DVT in AIS patients. We could not find literature directly addressing the relationship between the duration of EVT and neutrophil activation. However, it has been reported that thrombi from first‐pass recanalization patients, who achieve an mTICI score of 2c/3 after the first pass and usually require a shorter EVT time, have less extracellular DNA, a major component of NETs, than those from multiple‐pass recanalization patients, who need longer EVT time to obtain an mTICI score of 2c/3 (Vandelanotte et al. 2024). Moreover, EVT has the potential to damage endothelial cells and vessel walls, which is exacerbated by repeated stentriever passages (Hernandez et al. 2023; Teng et al. 2015). Whether the damaged endothelial cells and blood walls release tissue factors and subsequently activate the coagulation cascade in AIS patients needs to be further investigated. Thus, rapid recanalization of the occluded cerebral artery not only saves the ischemic brain tissue, but also prevents the formation of new thrombi in both the veins and arteries. However, it should be noted that the combination of lower extremity NIHSS score ≥ 2, plasma concentration of D‐dimer ≥ 1.62 mg/L, and EVT duration ≥ 66 min in our established nomogram has greater and more accurate predictive power than any single variable.

The association between the duration of EVT for the treatment of arterial thrombus in the brain and the prevalence of DVT in the lower extremities has piqued our interest in the relationship between arterial and venous thrombosis. As we have discussed above, neutrophil activation and hypercoagulability may establish a link between arterial and venous thrombosis, although further studies are needed for direct evidence. In fact, asymptomatic atherosclerotic lesions of the carotid artery have been reported to be related to spontaneous DVT in the legs (Prandoni et al. 2003). DVT has also been observed to increase the risk of subsequent myocardial infarction and stroke (Sorensen et al. 2007). Arterial and venous thrombosis share many common risk factors, such as age, obesity, hyperlipidemia and hypertension (Jerjes‐Sanchez 2005; Prandoni et al. 2003). Interestingly, both the antiplatelet medications commonly used for arterial thrombosis and the anticoagulants often used for venous thrombosis show therapeutic efficacy in arterial or venous occlusive disease (Koupenova et al. 2017). However, it should be noted that arterial and venous thrombosis have different pathophysiological mechanisms. The former occurs under high shear flow when platelet‐rich thrombi form around ruptured atherosclerotic plaques and damaged endothelium, whereas the latter occurs under low shear flow and usually around intact endothelial walls. Venous thrombi are rich in fibrin and encapsulate a large number of red blood cells in addition to activated platelets (Koupenova et al. 2017). Precise therapy may therefore be required for these two different thrombi.

Our study excludes infarct core size detected by CT scan as a predictor of DVT, despite its general correlation with stroke symptom severity. Whether the infarct results in lower limb weakness is also determined by the location of the infarction. For instance, a small infarct in the internal capsule can produce symptoms similar to those caused by a larger infarct in a brain lobe.

This study had some limitations. Firstly, it is based on retrospective data. The validity is inherently limited. In addition, most patients underwent only a single ultrasound examination during hospitalisation, with no subsequent follow‐up, which could miss the diagnosis of DVT in the following weeks after discharge and confound the results. The urgency of EVT in patients with AIS with significant vascular occlusion also precludes ultrasound confirmation of DVT prior to EVT. Furthermore, the development and internal validation of the model were limited to a single medical centre, with external validation performed at only one other centre. Therefore, prospective multi‐centre studies are needed to confirm the clinical utility of our prediction model for the occurrence of DVT.

## 5 Conclusion

Our study has developed and validated a nomogram incorporating lower limb NIHSS score, D‐dimer level, and the duration of EVT as effective predictors of DVT risk in patients with AIS after EVT. These findings not only improve our understanding of the predictors of DVT in this patient population but also suggest a broader, systemic predisposition to thrombosis that transcends the traditional arterial–venous divide. Addressing these risk factors through early identification and intervention may significantly improve outcomes in patients with AIS undergoing EVT.

## 6 Relevance to Clinical Practice

The developed nomogram provides a valuable clinical tool for predicting the risk of DVT after EVT. It allows healthcare professionals to identify high‐risk patients early and tailor preventive strategies effectively, which is crucial for improving patient outcomes and reducing the burden of complications associated with AIS.

## 7 Patient or Public Contribution

Patients or the public were not involved in the planning, conduct or reporting of our research. This approach was chosen due to the retrospective nature of our study, which relied on the analysis of existing medical records and did not involve direct patient interventions or feedback.

## Author Contributions

L.H. and T.‐W.P. contributed equally to this work as first authors, including conceptualisation, methodology, data curation, formal analysis and writing of the original draft. W.‐Y.Y. and Y.L. contributed equally as corresponding authors, providing oversight and critical revision of the manuscript for important intellectual content. F.W. and W.‐Z.W. provided funding support for the research and contributed to data analysis and interpretation. L.‐L.Y., W.‐Y.Q., and X.‐P.X. contributed to the conceptualisation, methodology and data interpretation.

## Conflicts of Interest

The authors declare no conflicts of interest.

## Supporting information


Table S1

Data S1

## Data Availability

The data supporting the study's findings are available from the corresponding author upon reasonable request.
