# Supplementary material for: Nomogram for Deep Vein Thrombosis Prediction Post‐Endovascular Thrombectomy in Acute Ischemic Stroke: A Retrospective Multicenter Observational Study
Source: J Clin Nurs. 2025 Apr 18;34(12):5293–305. doi: 10.1111/jocn.17786 (PMC12581100; doi:10.1111/jocn.17786)
Supplement: Supplementary file 1 — Table S1 [file JOCN-34-5293-s001.docx]

**Supplementary Table 1** Relationship between NIHSS score 24h post-EVT categories and DVT risk in the training cohort.

| **NIHSS** **Category** | **No. of DVT events/Participants** | **OR (95% Cl)** | |  | ***P*-value** | |  |
| --- | --- | --- | --- | --- | --- | --- | --- |
| Mild (0-4) | 5/103 | Ref |  | |  |  | |
| Moderate (5-15) | 33/115 | 7.88(3.02-20.55) |  | | <0.001 |  | |
| Severe (>15) | 36/74 | 18.56 (6.95-49.54) |  | | <0.001 |  | |

**Abbreviations**: DVT, deep vein thrombosis; EVT, endovascular thrombectomy; PRT, puncture-to-recanalization time; NIHSS, National Institutes of Health Stroke Scale.

**Supplementary Table 2** Relationship between predictors (PRT, D-dimer, and lower limb NIHSS score) and DVT risk, adjusted for NIHSS score 24h post-EVT categories in the training cohort.

| **Variable** | **OR** | **95% CI** | ***P*-value** |
| --- | --- | --- | --- |
| PRT | 2.14 | 1.15-4.07 | 0.018 |
| D-dimer | 3.29 | 1.17-6.20 | <0.001 |
| Lower limb NIHSS score ≥ 2 | 5.13 | 4.60-20.33 | 0.011 |

**Abbreviations**: DVT, deep vein thrombosis; EVT, endovascular thrombectomy; PRT, puncture-to-recanalization time; NIHSS, National Institutes of Health Stroke Scale.
